# The *Colletotrichum siamense* Hydrophobin CsHydr1 Interacts with the Lipid Droplet-Coating Protein CsCap20 and Regulates Lipid Metabolism and Virulence

**DOI:** 10.3390/jof8090977

**Published:** 2022-09-19

**Authors:** Na Wang, Jiyuan Wang, Jingwen Lu, Yu Liu, Yitao Xi, Miao Song, Xiaoling Guan, Zhigang Li, Xiao Li, Yu Zhang, Chunhua Lin, Weiguo Miao

**Affiliations:** Key Laboratory of Green Prevention and Control of Tropical Plant Diseases and Pests, Ministry of Education, College of Plant Protection, Hainan University, Haikou 570228, China

**Keywords:** *Colletotrichum siamense*, hydrophobin CsHydr1, lipid droplet-coating protein CsCap20, appressoriual turgor pressure, virulence

## Abstract

Previous studies of the lipid droplet-coating protein Cap20 in *Colletotrichum* show that it plays a key role in appressorium development and virulence. In this study, the hydrophobin CsHydr1, which contains a signal peptide of 19 amino acids and a hydrophobic domain (HYDRO), was shown to interact with CsCap20 in *Colletotrichum siamense.* The *CsHydr1* deletion mutant showed slightly enhanced mycelial growth, small conidia, slow spore germination and appressoria formation, cell wall integrity and virulence. Like CsCAP20, CsHydr1 is also localized on the lipid droplet surface of *C. siamense*. However, when CsCap20 was absent, some CsHydr1 was observed in other parts. Quantitative lipid determination showed that the absence of either CsHydr1 or CsCap20 reduced the content of lipids in mycelia and conidia, while the effect of CsCap20 was more obvious; these results suggest that an interaction protein CsHydr1 of CsCap20 is localized on the lipid droplet surface and involved in lipid metabolism, which affects appressorium formation and virulence in *C. siamense*.

## 1. Introduction

*Colletotrichum* is a large ascomycete genus that includes numerous important plant pathogenic species and species complexes that infect a wide variety of hosts and cause anthracnose on cereals, legumes, fruit trees, and vegetables [1]. Among them, *C. siamense* was classified into a main phylogenetic clade within the *C. gloeosporioides* species complex and causes anthracnose disease in a wide range of plants. In China, *C. siamense* is reported to be the major pathogenic species of rubber tree *Colletotrichum* leaf disease (CLD) in the field [2,3,4], which is also the main pathogenic species of anthracnose in many other tropical or subtropical crops, such as *Mangifera indica* [5], *Litchi chinensis* [6], *Camellia chrysantha* [7], *Zizyphus mauritiana* [8], *Alocasia macrorrhiza* [9], *Coffea arabica* [10], and so on. The study of the molecular mechanisms of *C. siamense* pathogenesis may provide a foundation for formulating prevention and control strategies.

In the pathogenic process of many phytopathogenic fungi, including *Colletotrichum* spp., conidia attach to the leaf surface and produce germ tubes, which extend and differentiate into bulbous melanized appressoria [11,12]. Mature appressoria with high turgor pressure are essential to penetrate the leaf surface [13,14]. Lipids are crucial for fungal virulence, from spore germination to the development of mature appressoria. Lipid bodies move into the appressorium during maturation, where they are degraded by triacylglycerol lipase during turgor generation [13,15]. Previous studies have shown that perilipin, a lipid droplet-associated protein, is localized to the lipid droplet surface and is essential to increase lipid flux into or out of lipid droplets [16]. Perilipin or its homologue is reported to be involved in lipid diseases in mammals and insects. For example, perilipin variants are involved in obesity or leanness in mice, humans, and fruit flies [17,18,19]. In insect pathogenic fungi, it has also been confirmed that the perilipin homologue MPL1 in *Metarhizium anisopliae* affects lipid metabolism, appressorial turgor pressure and virulence [20]. In a previous study, the *Cap20* gene was cloned and confirmed to be involved in the virulence of *C.* *gloeosporioides* in avocado and tomato fruits [21]. Moreover, our group has demonstrated that the CsCap20 protein is a perilipin homologue and is involved in the regulation of the number of lipid droplets and appressorium turgor in *C.* *siamense* from rubber tree [22]. To better understand the regulatory mechanism of CsCap20, interacting proteins, including a hydrophobin, were screened by a yeast two-hybrid system [23].

Hydrophobins (HPs) are small secreted amphiphilic proteins comprising 82–170 amino acids that are only found in filamentous fungi [24,25,26]; they are hydrophobic and can self-assemble into amphiphilic layers at hydrophilic/hydrophobic or air/water interfaces, allowing fungi to grow into the air from an aqueous environment by reducing the water surface tension [27,28]. Hydrophobins have been proposed to participate in many developmental processes in fungi, including hyphal growth, attachment to hydrophobic surfaces, sporulation, and sexual development [24,29,30,31]. Some hydrophobins are reported to be involved in fungal virulence, such as MPG1 and MPH1 in *Magnaporthe grisea* [32,33,34,35], Hyd1 and Hyd2 in *Beauveria bassiana* [36], and FgHyd2, FgHyd3 and FgHyd4 in *Fusarium graminearum* [31]. Nevertheless, there are no reports on the relationship between hydrophobins and lipid droplet-coating proteins or lipid droplets.

Here, we characterized the function of the hydrophobin CsHydr1, which affects spore germination, appressorium formation and virulence. Additionally, we demonstrated that CsHydr1 interacts with CsCap20, localizes on the lipid droplet surface and influences the lipid content in *C. siamense*; these findings could enrich the understanding of the pathogenic mechanism of hydrophobin in fungi.

## 2. Materials and Methods

### 2.1. Fungal Strains and Culture Conditions

The *C. siamense* HN08 strain was used as a wild type (WT) in this study [23,37]. The gene deletion mutant Δ*CsCap20* and protein localization strain Cap20-GFP were constructed by our research group previously [22]. The gene deletion mutant Δ*CsHydr1*, the complementary strain Δ*CsHydr1* (*Hydr1*) and the protein subcellular localization strains *WT-CsHydr1-myc* and Δ*CsCap20-CsHydr1-myc* were constructed in this study. For the collection of conidia, hypha was placed and cultured on potato dextrose agar medium (PDA) under continuous fluorescent light for 3–5 days at room temperature. For collection of mycelia, hypha was cultured in liquid complete medium (CM) plates [38].

### 2.2. CsHydr1 Gene Cloning and Sequence Analysis

The CsCap20 protein of *C. siamense* was used as a bait protein to screen the *C. siamense* yeast cDNA library to obtain partial sequences of *CsHydr1* [23]. The *CsHydr1* coding regions and upstream and downstream sequences were obtained from the genome database and transcriptome database of HN08 by using a local BLAST search. The primers hydr-F and hydr-R were designed for amplification of the whole coding sequences of *CsHydr1* DNA and cDNA. Then cDNA sequence was transformed into an amino acid sequence and analyzed in Modular Architecture Research Tool (SMART, http://smart.embl-heidelberg.de/, accessed on 15 May 2022). The phylogenetic tree was constructed by the maximum likelihood method in MEGA6 [39]. The phylogenetic tree was supported with 1000 bootstrap values.

### 2.3. Protein Secretion Verification of CsHydr1

*CsHydr1* (containing full-length cDNA sequences) and *CsHydr1*^Δ^*^sp^* (signal peptide removed, containing only 119 C-terminal amino acid residue sequences) were amplified by Mi-H1-F/Mi-H1-R and Mi-sp-H1-F/Mi-sp-H1-R and ligated to the pSUC2 plasmid using double digestion (*Eco*R Ⅰ and *Xho* Ⅰ) methods, respectively. Primers are presented in Table S1. Then, the recombinant plasmids pSUC2-CsHydr1 and pSUC2-CsHydr1^Δ^*^sp^* were transformed into yeast strain YTK12. Thereafter, plate screening and TTC (2,3,5-triphenyl tetrazolium chloride) assays were performed as described by Li et al. [40].

### 2.4. Targeted Disruption of CsHydr1 and Complementation

The vector PCX62-S with the sulfonylurea resistance gene (*ILV1*) was constructed previously in our lab and used as a structure plasmid. The knockout vector PCX62-S-CsHydr1 was constructed by double digestion and homologous recombination methods as described [38]. The schematic diagram is shown in Figure S1a. Approximately 600 bp sequences upstream and downstream of the *CsHydr1* gene were amplified using Hydr-U-F/Hydr-U-R and Hydr-D-F/Hydr-D-R primers, which added the linkers individually (Table S1). Then, the upstream fragment digested by *Eco*R I and *Hin*d III were ligated to the *N*-terminal end of the *ILV1* gene by T_4_ ligase to obtain the recombinant plasmid PCX62-S-U. The downstream fragment and the PCX62-S-U vector were digested with *Bam*H I, which were ligated to the C-terminus of *ILV1* using yeast homologous recombination. And the correct vector PCX62-S-CsHydr1 was confirmed by sequencing. Finally, plasmid PCX62-S-CsHydr1 was transformed into protoplasts of HN08 and screened at DCM with 100 μg/mL sulfonylurea as described previously [22,37,38]. *CsHydr1* deletion mutant was confirmed by PCR with several pairs and Southern blot analysis as shown in Figure S1. Southern hybridization was performed by manual operation of the DIG High Prime DNA Labeling and Detection Starter Kit I (Roche, Basel, Switzerland).

A gene complementation vector was constructed using the PXY203 vector (containing the RP27 promoter and the hygromycin transferase gene *HPH*). The primers PXY203-Hydr1F/R were used to amplify the coding sequence of *CsHydr1* with a homologous linker. Both of PXY203 vector and *CsHydr1* amplified fragment had been digested with *Xho* I and they were co-transferred into yeast XK1-25 for yeast homologous recombination, resulting in PXY203-CsHydr1 plasmid. Then, the plasmid PXY203-CsHydr1 was introduced into the Δ*CsHydr1* mutant protoplasts, and transformants were screened by PDS medium with hygromycin as [38] and verified by PCR, which resulted in complementary strain Δ*csHydr1 (Hydr1)*. The expression of *CsHydr1* in the Δ*csHydr1 (Hydr1)* was followed by the confirmation with RT-PCR using the RT-Hydr1-F/RT-Hydr1-R primer pair. The expression of the *CsHydr1* gene relative to an internal control housekeeping *ACT* gene.

### 2.5. Validation of Protein Interactions between CsHydr1 and CsCap20

#### 2.5.1. Yeast Two-Hybrid Analysis

The fragment of *CsHydr1*^Δ*sp*^ was cloned into the plasmid pGBKT7, resulting in pGBKT7-CsHydr1^Δ*sp*^. The whole *CsCap20* fragment was cloned into the plasmid pGADT7, and pGADT7-CsCap20 was constructed (primers are listed in Supplementary Table S1). Then, pGBKT7-CsHydr1^Δ*sp*^ and pGADT7-CsCap20 were co-transformed into yeast Y2H. Next, yeast cells were plated on SD/-Trp-Leu plates and validated by PCR using pGBKT7-F/R and pGADT7-F/R primers. Yeast containing plasmids pGBKT7-CsHydr1^Δ*sp*^ and pGADT7-CsCap20 were dropped on SD/-Trp-Leu and SD/-Trp-Leu-His-Ade plates, pGBKT7-p53+pGADT7-T served as a positive control, and pGBKT7-lam+pGADT7-T was a negative control and then incubated at 30 °C for 3 d to observe yeast growth.

#### 2.5.2. HIS Pull-Down Assay

The recombinant plasmids pGEX-6p-CsHydr1^Δ*sp*^ and pET32a-CsCap20 were constructed by the double digestion method (*Bam*H Ⅰ and *Eco*R Ⅰ) (primers are listed in Table S1). His-Cap20 and GST-CsHydr1 fusion proteins were expressed in *E. coli* BL21. Prokaryotic expression, western blot analysis and pull-down assays were performed according to the method described by Wang et al. [23].

#### 2.5.3. Coimmunoprecipitation (Co-IP) Assays

The vector pFL21 and the *CsHydr1* fragment (*C*-terminus ligated the myc sequence) were digested with *Xho* I and transferred to yeast XK1-25 for yeast homologous recombination. The recombinant plasmid pFL21-CsHydr1 with *CsHydr1*, myc tag and *hph* (hygromycin resistance gene) was obtained. Plasmid pCB1532-Cap20-GFP was constructed previously [22]. The plasmids pFL21-CsHydr1 and pCB1532-GFP-Cap20 were co-transformed into HN08, and pFL21-CsHydr1 was transformed into HN08 as a control. The transformant coexpressing CsHydr1-myc and GFP-CsCAP20 was selected from the wild-type strain on a medium containing both 100 μg/mL sulfonylurea and 200 μg/mL hygromycin. In addition, the control transformant expressing CsHydr1-myc only was obtained from a medium supplemented with 200 μg/mL hygromycin. Finally, the total proteins of these two kinds of transformants were extracted, and the interactions between CsHydr1-myc and CsCap20-GFP in *C. siamense* were detected by immunoprecipitation and western blotting as described by Wang et al. [23].

### 2.6. Phenotype Analysis

The spore suspensions of the HN08, Δ*csHydr1* and Δ*csHydr1*(*Hydr1*) strains were prepared according to Song et al. [38]. Then, 20 μL spore suspensions (1 × 10^5^ spores/mL) were dropped onto the surface of glass slides, and spores and appressoria were examined under the microscope after incubation at 28 °C according to Song et al. [38]. One hundred spores were counted for each strain. Three independent experiments were performed.

To assay the effects of different stress conditions on tested strains, 10 μL of spore suspension (1 × 10^5^ spores/mL) was inoculated on PDA plates supplemented with sorbitol (1 M), NaCl (1 M), and Congo Red (CR, 100 μg/mL). Colony diameters were measured and photographed after incubation for 5, 7, and 9 days at 28 °C. PDA plates were used as controls. All experiments were carried out in triplicate.

To test the virulence, 20 μL of spore suspensions (1 × 10^5^ spores/mL) of tested strains were dropped on the detached tender leaves with or without wounded as [38] described. Three technical replicated were used for each treatment, and 30 leaves were inoculated for each treatment. The disease lesions were measured and photographed 3 days after inoculation.

### 2.7. Scanning Electron Microscopy

10 μL of spore suspensions (5 × 10^5^ spores/mL) of the HN08, Δ*csHydr1* and Δ*csHydr1*(*Hydr1*) strains were prepared and dropped onto the surface of silicon wafers, respectively. After the droplets were blown dry, the samples were attached to the sample stage with double-sided tape (to avoid contact with the viewing surface of the sample). The Au sputterer was coated to observe the spore surface properties using field emission scanning electron microscopy (Thermo Scientific, Thermo Fisher Scientific, Waltham, MA, USA).

### 2.8. Immunofluorescence, NR and DiD Staining

The plasmid pFL21-CsHydr1 was transformed into HN08 and Δ*csCap20*, and the subcellular localization strains WT-CsHydr1-myc and Δ*csCap20-CsHydr1-myc* were obtained. 20 μL of spore suspension (1 × 10^5^ spores/mL) was dropped onto the surface of glass slides, and the localization of CsHydr1 was observed at different stages.

Immunofluorescence staining: Slides were washed once with 1× PBS solution, fixed at room temperature for 30 min (4% paraformaldehyde fix solution, Sangon Biotech, Shanghai, China), washed three times with 1× PBS solution, and subjected to a closed reaction with 1% bovine serum albumin (BSA, Sangon Biotech, Shanghai, China) solution for 1 h at room temperature. The primary antibody reaction with c-Myc (concentration 0.5 µg/mL, Beyotime, Shanghai, China) was performed for 1 h and washed three times with 1× PBS solution. Then, the fluorescent secondary antibody was immunoprecipitated for 1 h in the dark and finally washed with 1× PBS solution three times. The fluorescence channel of GFP was used for observation.

Nil Red staining: After immunofluorescence staining, 100 µL of Nile Red staining solution (concentration 1 µg/mL, Solarbio, Beijing, China) was added dropwise to the slide and stained for 6 min at room temperature under dark conditions, followed by three washes with 1× PBS solution. The fluorescence channel of RFP was used for observation.

DiD staining: After immunofluorescence staining, 100 μL of DiD staining solution (KeyGen Biotech, Nanjing, China) was added dropwise to the slides and stained for 30 min at 37 °C in the dark, followed by three washes with 1× PBS solution. The fluorescence of GFP and RFP were observed by using a Nikon confocal microscope (Nikon, Tokyo, Japan).

### 2.9. Lipid Quantitation

To determine the lipid content, the mycelia of the tested strains were collected from CM for 3 days incubation and conidia were collected from PDA plates for 5 days inoculation by filtration and freeze-dried. 0.5 mL of spore suspension (1–1.5 × 10^8^ spores/mL) and 0.2 g of mycelia of tested strains were prepared and added to separate glass tubes. Quantitative determination of total lipids was assessed by the phosphoric acid-vanillin method according to Wang et al. [20]. A standard curve was generated with triolein (Sangon Biotech, Shanghai, China). Differences in lipid content between treatments were compared using Duncan’s analysis of variance (IBM SPSS Statistics 25, Armonk, NY, USA).

## 3. Results

### 3.1. The C. siamense CsHydr1 Protein Is a Secreted Protein and Belongs to Class I Hydrophobin

**Figure 1:**
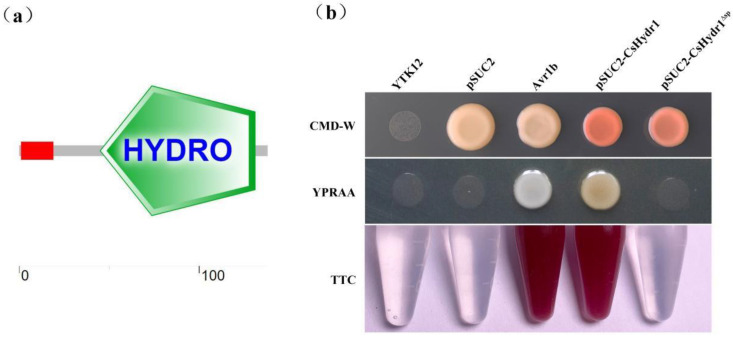
Protein structure and secretion of CsHydr1 in *C. siamense*. (**a**) SMART analysis of CsHydr1 protein structure. The red region is the signal peptide. The green region is the HYDRO domain; (**b**) Functional validation of the signal peptide of CsHydr1 using the yeast invertase secretion assay. The full-length and *C*-terminal amino acid residue sequences without signal peptide (*CsHydr1*^Δ*sp*^) of the *Cs*Hydr1 gene were fused into the pSUC2 vector and transformed into the yeast YTK12 strain. The signal peptide of a known secreted protein Avr1b was used as a positive control. The untransformed YTK12 and YTK12 carrying the pSUC2 vector were used as a negative control. Yeast growth on the CMD-W medium confirmed that the vector had transformed into the yeast cell. The growth in the YPRAA medium and color change of TTC confirmed invertase secretion.

Our previous study showed that hydrophobin (ELA31811.1) is a putative CsCAP20-interacting protein identified by the Y2H system [23]. To confirm their interaction relationship and the biological significance of this hydrophobin, the coding sequence of this hydrophobin was obtained via RT-PCR from the *C. siamense* HN08 strain; this gene putatively encodes a 138-amino-acid protein containing a signal peptide consisting of 19 amino acids at the *N*-terminus and a HYDRO domain at the *C*-terminus of the protein (Figure 1a); its amino acid sequence showed 99% identity to hypothetical hydrophobin of *Colletotrichum* spp. (KAF4805904.1, XP_036491983.1, XP_031884570.1), but low identity with known hydrophobin of other fungi (Figure S2); its cysteine pattern is CN{7}CCN{36}CN{15}C{5}CC{16}C (where N signifies any other amino acid than cysteine). According to Wösten’s classification criteria for hydrophobins [41,42], this protein is classified as type I hydrophobin. We named it *CsHydr1* and deposited the sequence in GenBank (Accession No. MN166623).

Since protein CsHydr1 carries a predicted *N*-terminal signal peptide, we determined its secretory function using the yeast secretion system [43,44]. The full-length (138 amino acids) and *C*-terminal 119 amino acid residue sequences (*CsHydr1*^Δ*sp*^) of the *CsHydr1* gene were fused in vector pSUC2 and then transformed into yeast strain YTK12. The results showed that the full-length sequences of *CsHydr1* and *Avr1b* (positive control) allowed YTK12 to grow on the YPRRA medium (Figure 1b). Meanwhile, the strain transformed with the full-length sequences of *CsHydr1* displayed secreted invertase enzyme activity that catalyzed the reduction of TTC to a red-colored triphenyl formazan (Figure 1b). In contrast, the *CsHydr1*^Δ*sp*^ and negative controls (including the untransformed YTK12 strain and YTK12 carrying the pSUC2 vector) did not allow YTK12 strains to grow on YPRRA, and the TTC-treated culture filtrates remained colorless (Figure 1b). Therefore, CsHydr1 is a secreted protein, and its *N*-terminal signal peptide is required for its secretion.

### 3.2. Targeted Deletion of the CsHydr1 Gene and Complementation

For gene deletion, 80 transformants were screened by resistance to sulfonylurea in total. Among them, a Δ*csHydr1* mutant was confirmed as a *CsHydr1* gene deleted mutant by PCR amplification, sequencing and Southern hybridization analysis (Figure S1). The internal sequence of the *CsHydr1* gene could not be amplified by the primers Hydr-F/Hydr-R, and the products were amplified by the primers Hydr-U-Ou-F/S2R and S1F/Hydr-D-Ou-R in the Δ*csHydr1* mutant but not in the wild type, which indicated that *CsHydr1* was replaced by the *ILV1* gene (Figure S1a). In addition, the PCR products from both Δ*csHydr1 (*4240 bp) and the wild type (1943 bp) using the primers Hydr-U-Ou-F/Hydr-D-Ou-R were confirmed by sequencing (Figure S1a), which showed that the 520-bp open reading frame of the CsHydr1 gene was replaced by the 2817-bp fragment of the *ILV1* gene, as shown in Figure S1a. The hybridization result showed that only one copy of the *ILV1* gene in the Δ*csHydr1* mutant genomic DNA (Figure S1c). Therefore, Δ*csHydr1* was indeed a CsHydr1 null mutant.

Then, the recombination sequence of the open reading frame of the *CsHydr1* gene with the RP27 promoter was reintroduced into the Δ*csHydr1* mutant, which resulted in the complementation strain Δ*csHydr1*(*Hydr1*). The Δ*csHydr1*(*Hydr1*) strain was verified by PCR and RT-PCR (Figure S1d,e).

### 3.3. CsHydr1 Deletion Mutants Showed Slightly Increased Mycelial Growth, Thin Conidia, Increased Spore Germination Rate and Appressorium Formation Rate

**Figure 2:**
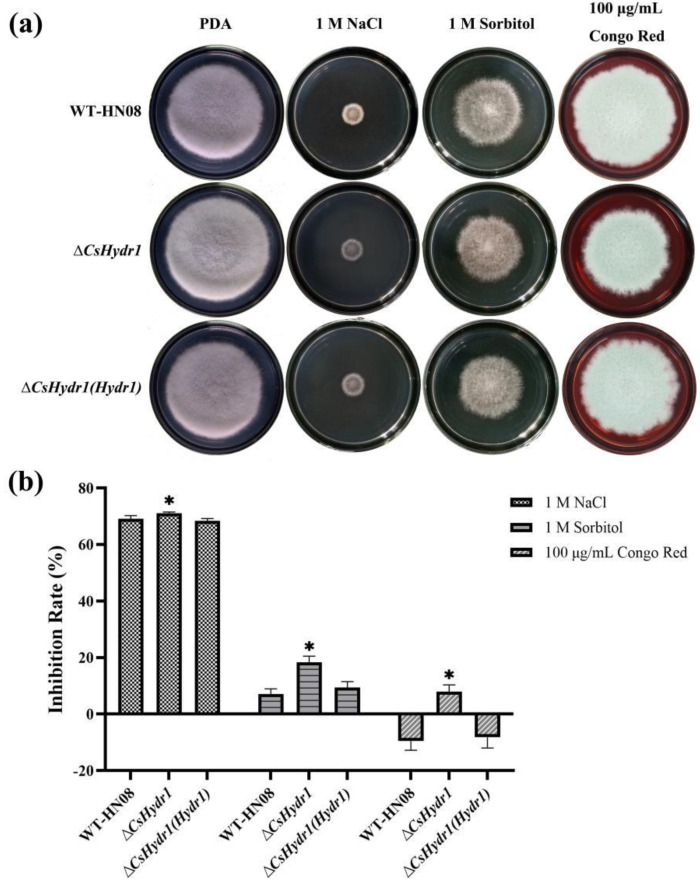
Response of *CsHydr1* mutants to various stress conditions. (**a**) Growth of the tested strains on PDA plates supplemented with 1 M NaCl, 1 M sorbitol and 100 μg/mL Congo Red for 7 days; (**b**) Growth inhibition rate of the tested strains under various stresses. The inhibition rate of mycelial growth was calculated using the following formula: Inhibition rate (%) = [(control diameter – treated diameter)/(control diameter × 100%)]. Each treatment had three replicates. Error bars represent the standard deviations. * indicated significant differences within each measurement group (*p* < 0.1, One-way Anova and Duncan’s test).

The mycelial growth of the wild-type, Δ*csHydr1* mutant, and Δ*csHydr1*(*Hydr1*) was determined under various stress conditions (Figure 2). No significant difference in the morphology of the three colonies was observed. However, the colony diameter was 6.71 ± 0.23 cm after incubation on a PDA medium for 7 days at 28 °C, and those of the wild type and Δ*csHydr1*(*Hydr1*) were 5.96 ± 0.14 cm and 6.03 ± 0.30 cm, respectively. The growth inhibition rate of Δ*csHydr1* exposed to 100 μg/mL Congo Red, 1 M sorbitol and 1 M NaCl was, on average 7.64%, 18.81% and 70.95% higher than that of the wild-type and Δ*csHydr1*(*Hydr1*) strain, respectively. The results indicated that the *CsHydr1* gene affects mycelia growth, cell wall integrity and osmotic sensitivity slightly.

**Figure 3:**
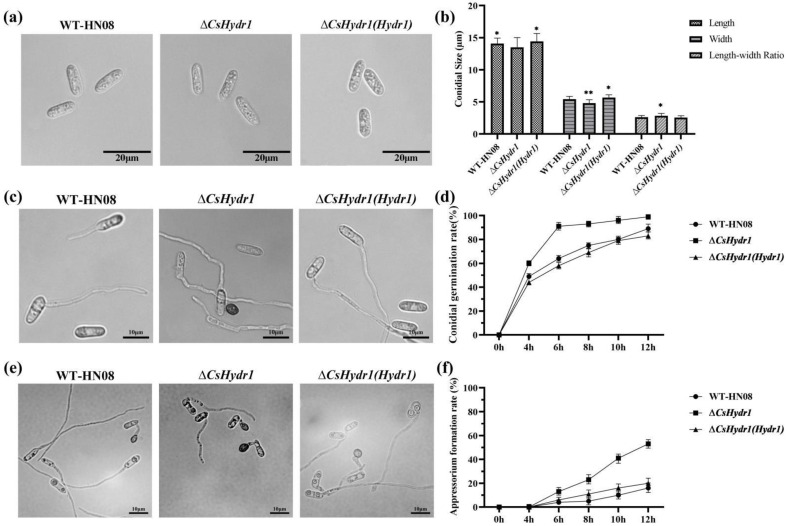
Comparison of conidial size, germination rate and appressorium rate of wild-type HN08, Δ*csHydr1* mutant, and Δ*csHydr1*(*Hydr1*) strain. (**a**,**b**) are the morphology, and conidial size of the tested strains; (**c**,**d**) are morphology and conidial germination rates of the tested strains; (**e**,**f**) are morphology and appressorium formation rates of the tested strains. * indicated significant differences within each measurement group (* *p* < 0.1, ** *p* < 0.01, One-way Anova and Duncan’s test).

The conidia sizes were measured, and the results showed that the sizes of the Δ*csHydr1* mutant were (13.52 ± 0.32) × (4.80 ± 0.13) µm, and those of the wild-type and Δ*csHydr1*(*Hydr1*) were (14.07 ± 0.08) × (5.41 ± 0.13) µm and (14.43 ± 0.25) × (5.66 ± 0.07) µm (Figure 3a), respectively. The length-width ratio of Δ*csHydr1* was slightly larger than that of the other two strains (Figure 3b). The data showed that the Δ*csHydr1* mutant had slightly thin conidia.

The rate of spore germination and appressorium formation was tested from 4 h to 12 h after inoculation; it showed that the spore germination rate of Δ*csHydr1* was 91% at 6 h, while those of the wild type and Δ*csHydr1*(*Hydr1*) were 64% and 58%, respectively (Figure 3c,d). The appressorium rate of Δ*csHydr1* was 57%, while those of the wild type and Δ*csHydr1*(*Hydr1*) were 16% and 17% at 12 h after inoculation, respectively (Figure 3e,f). Which meant the spore germination rate and the appressorium rate of the Δ*csHydr1* mutant was higher than those of the wild type and Δ*csHydr1*(*Hydr1*).

**Figure 4:**
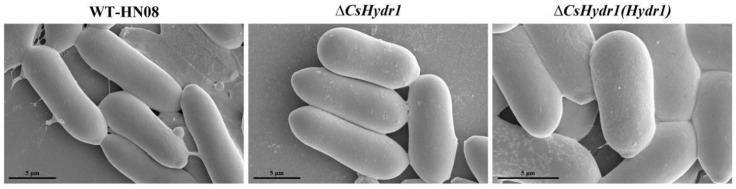
Electron microscopy of spore surfaces of HN08, Δ*csHydr1* mutant, and Δ*csHydr1*(*Hydr1*) strain. Bars, 5 µm.

Since many hydrophobins are involved in spore superficial rodlet layer formation [25,41,45], we observed the rodlet layer present in the spores of tested strains by scanning electron microscopy. The results showed that the surface of conidia of all three strains was smooth, and no rodlet layers were observed (Figure 4); it was indicated that the function of *CsHydr1* on the formation of rodlets was not obvious. In conclusion, these data indicated that the loss of *CsHydr1* slightly affected the mycelial growth, conidia size, spore germination and appressorium formation rates, but not the rodlet layer of conidia.

### 3.4. CsHydr1 Is Responsible for the Full Virulence of C. siamense

**Figure 5:**
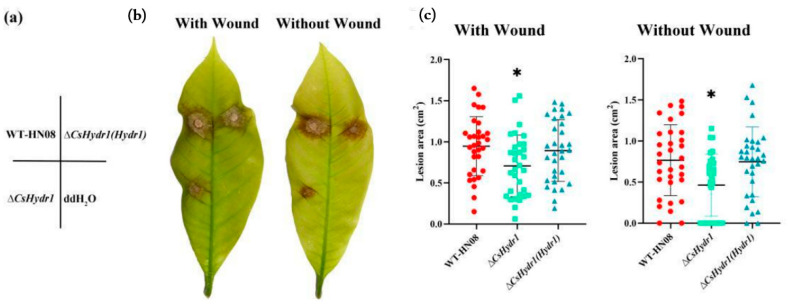
Virulence assays on rubber tree leaves. (**a**) Schematic diagram of the virulence assays of the tested strains. The rubber tree leaves were inoculated with 20 μL of conidial suspension (1 × 10^5^ spores/mL) of tested strains through unwounded and wounded ways; The symptoms (**b**) and dot plot analysis of the lesion areas (**c**) were shown at 5 days after inoculation. Thirty leaves were inoculated per treatment (* *p* < 0.1, according to one-way ANOVA and Duncan’s test; the error bar shows the standard deviation value).

The pathogenicity of the wild-type HN08 strain, Δ*csHydr1* mutant, and strain Δ*csHydr1*(*Hydr1*) were comparable on healthy light green rubber tree leaves that were wounded or nonwounded. The lesion area and infection rate were measured after 5 days of inoculation (Figure 5). On the wounded leaves, all three strains caused symptoms, and the diseased area caused by Δ*csHydr1* (mean 0.71 ± 0.37 cm^2^) was slightly smaller than those caused by the HN08 strain (mean 0.95 ± 0.36 cm^2^) and Δ*csHydr1*(*Hydr1*) strain (mean 0.89 ± 0.38 cm^2^). On the nonwounded leaves, the infection rate of Δ*csHydr1* (65.63%) was significantly lower than those of the HN08 strain (93.75%) and Δ*csHydr1*(*Hydr1*) strain (93.75%), and the diseased area caused by Δ*csHydr1* (mean 0.46 ± 0.38 cm^2^) was also slightly smaller than those of HN08 strain (mean 0.77 ± 0.43 cm^2^) and Δ*csHydr1*(*Hydr1*) strain (mean 0.75 ± 0.42 cm^2^). The results illustrated that the lack of *CsHydr1* affects fungal virulence to some extent.

### 3.5. The Protein CsHydr1 Interacts with CsCap20

**Figure 6:**
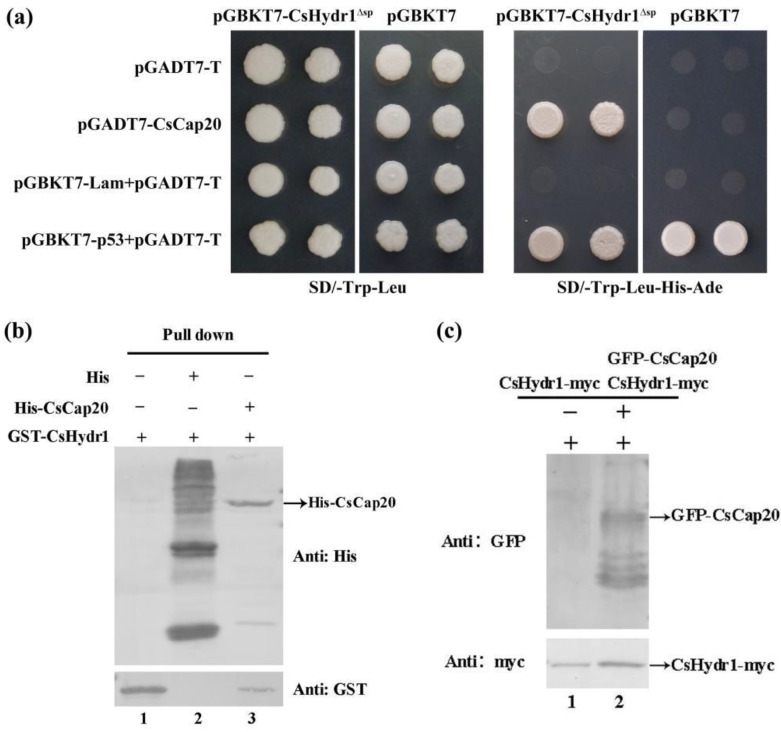
Identification of the interaction between CsHydr1 and CsCap20. (**a**) Confirmation of the interaction between pGADT7-CsCap20 and pGBKT7-CsHydr1^Δ*sp*^ by yeast two-hybrid assay. pGBKT7-p53+pGADT7-T is the positive control; pGBKT7-Lam+pGADT7-T is the negative control; (**b**) HIS pull-down results between His-CsCap20 and GST-CsHydr1. Lane 1 is positive control of GST-CsHydr1; Lane 2 is negative control 6 × His null protein and GST-CsHydr1 protein; Lane 3 is His-CsCap20 protein and GST-CsHydr1 protein; (**c**) Co-IP results between GFP-CsCap20 and CsHydr1-myc. Lane 1 is the control protein from the transformant expressing CsHydr1-myc only; Lane 2 is the protein from the transformant expressing both GFP-CsCap20 and CsHydr1-myc. +, protein added, −, protein not added.

To confirm the interaction relationship between CsHydr1 and CsCap20, a yeast two-hybrid system assay was conducted firstly. The results are shown in Figure 6a. All yeast colonies cotransformed with pGBKT7-CsHydr1^Δsp^ and pGADT7-CsCap20, negative control pGBKT7-Lam and pGADT7-T, or positive control pGBKT7-p53 and pGADT7-T grew on SD/-Trp/-Leu. Only yeast colonies co-transformed with pGBKT7-CsHydr1^Δsp^ and pGADT7-CsCap20, and the positive controls pGBKT7-p53 and pGADT7-T could grow on SD/-Trp/-Leu/-His/-Ade (Figure 6a); this result preliminarily verified the interaction between CsHydr1^Δsp^ and CsCap20.

Secondly, recombinant His-CsCap20 protein and GST-CsHydr1 protein were purified and subjected to a His pull-down experiment. The experimental group comprised His-CsCap20 protein and GST-CsHydr1 protein. The positive control group comprised GST-CsHydr1, and the negative control group comprised HIS protein and GST-CsHydr1. The results confirmed that the CsHydr1 protein interacts with CsCap20 (Figure 6b).

Finally, the interaction between CsHydr1 and CsCap20 in vivo was further verified by the Co-IP method. The transformant coexpressing CsHydr1-myc and GFP-CsCap20, and the control transformant expressing CsHydr1-Myc only were obtained. The total proteins of these transformants were extracted and detected by immunoprecipitation and western blot. The results showed that the experimental group CsHydr1-Myc successfully pulled down GFP-CsCap20 from total protein, whereas no interaction with GFP-CsCap20 in the control was observed (Figure 6c). Thus, the interaction of CsHydr1 with CsCap20 was verified in vitro and in vivo.

### 3.6. Protein CsHydr1 and CsCap20 Both Localize on Lipid Droplet Surface

**Figure 7:**
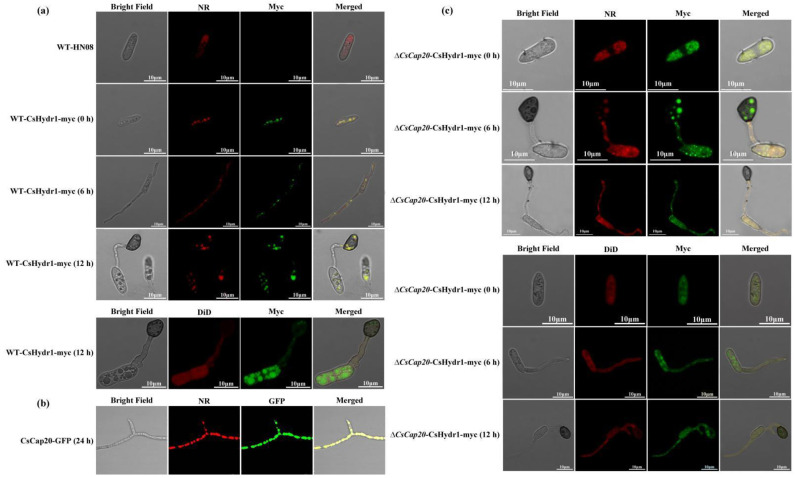
Subcellular localization of the CsHydr1 protein in WT and Δ*csCap20*. (**a**) Protein CsHydr1 localization in WT. (**b**) Protein CsCap20 localization in WT HN08. (**c**) Protein CsHydr1 localization in Δ*csCap20*. WT-CsHydr1-myc, the fusion protein CsHydr1-myc expressing in wild-type HN08; CsCap20-GFP, the fusion protein expressing in wild-type HN08; Δ*csCap20*-CsHydr1-myc, the fusion protein CsHydr1-myc expressing in the Δ*csCap20* mutant; Myc, immunofluorescence staining using antibodies against c-Myc; NR, Nile red staining; DiD, DiD staining.

Immunofluorescence (IF) staining was performed to measure the subcellular localization of the CsHydr1 protein. The recombination plasmid pFL21-CsHydr1 containing a myc tag was constructed. Then, it was introduced into wild-type HN08 and produced the transformant WT-CsHydr1-myc. The transformant WT-CsHydr1-myc underwent immunofluorescence staining using antibodies against c-Myc and analyzed for Myc, NR and DiD signals using epifluorescence microscopy. The results showed that CsHydr1 localized on the lipid droplets in conidia, germ tubes and appressoria (Figure 7a); this subcellular localization of CsHydr1 was consistent with a previous report of CsCap20 (Figure 7b) [22].

CsHydr1 interacted with CsCap20, and both the two proteins localized on the lipid droplets. We wanted to determine whether CsCap20 influenced the subcellular localization of CsHydr1. We introduced the plasmid pFL21-CsHydr1 into Δ*csCap20* and resulted in the transformant Δ*csCap20-CsHydr1-myc*. Similar to WT-CsHydr1-myc, the CsHydr1 protein is mainly localized on lipid droplets. However, we could clearly observe that some CsHydr1 localized on other parts in addition to lipid droplets when CsCap20 was absent (Figure 7c); these data suggested that CsHydr1 mainly localized to intracellular lipid droplets, and protein CsCap20 was helpful for the localization of CsHydr1 on lipid droplets in *C. siamense*.

### 3.7. Deletion of the CsHydr1 or CsCap20 Genes Decreased the Lipid Content in C. siamense

**Figure 8:**
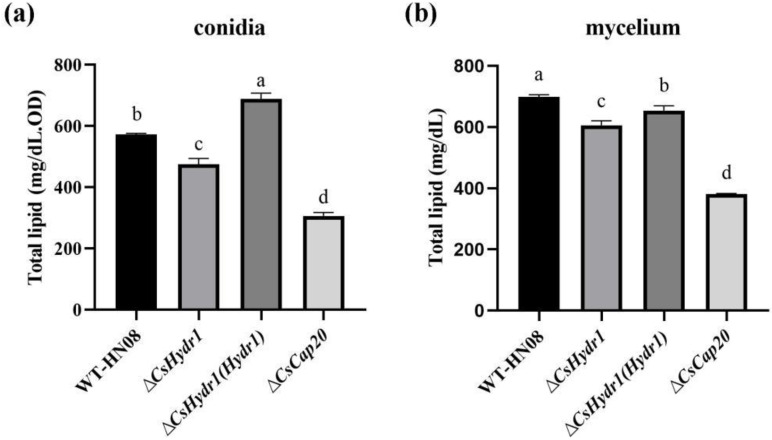
Lipid content relationship of CsHydr1 and CsCap20. Quantification of lipids from conidia (**a**) and mycelia (**b**) in HN08, the Δ*csHydr1* mutant, the Δ*csHydr1*(*Hydr1*) strain and the Δ*csCap20* mutant. Different letters (a, b, c, d) represent significant difference.

A previous study showed that Cap20 of *C. gloeosporioides* and the homologous protein MPL1 of *M. anisopliae* regulated lipid metabolism and decreased the total lipids in a gene deletion mutant [20,22]. Based on both CsHydr1 and CsCap20 being localized to lipid droplets, we also quantitatively determined the lipid content on conidia and mycelia of all of the wild types, Δ*csHydr1* and Δ*csCap20* (Figure 8). The results showed that the lipids on the conidia and mycelia of HN08 were 572.92 ± 3.36 mg/dL. OD and 698.24 ± 3.95 mg/dL, respectively. Compared with HN08, the lipid content was significantly decreased in both Δ*csHydr1* (475.87 ± 18.17 mg/dL. OD in conidia, 605.96 ± 3.45 mg/dL in mycelium) and Δ*csCap20* (299.71 ± 1.70 mg/dL. OD in conidia, 381.58 ± 2.17 mg/dL in mycelium); these data indicated that the absence of *CsHydr1* or *CsCap20* both reduced the content of lipids in mycelia and conidia, while the effect of CsCap20 was more obvious.

## 4. Discussion

Filamentous fungi generally contain multiple hydrophobin genes, which play important roles in fungal growth, development and environmental communication [26,46,47,48]. In this study, we cloned the class Ⅰ hydrophobin family member CsHydr1 in *C. siamense* from *Hevea brasiliensis* and confirmed that CsHydr1 was involved in the morphogenesis of hyphae and conidia, affecting cell wall integrity and pathogenicity in *C. siamense*. Furthermore, we demonstrated that hydrophobin CsHydr1 interacted with the lipid droplet-associated protein CsCap20, which is involved in pathogenicity, localized on the surface of lipid droplets and involved in regulating the content of lipid droplets and affecting pathogenicity.

Hydrophobins are small, secreted proteins and amphiphilic proteins that can self-assemble into monolayers on hydrophobic: hydrophilic interfaces and can form a rodlet layer to be used for surface coatings [49]. Hydrophobins are capable of altering the surface tension and hydrophobicity, which affect the growth of hyphae, including Trhfb3 in *T. reesei* [50], SC3 in *S. commune* [51], Hyd1 and Hyd3 in *C. rosea* [52], and most hydrophobins in *Coriolopsis trogii* [53]. The protein CsHydr1 in *C. siamense* was identified as a secreted protein, and a gene knockout mutant also led to a slight increase in the colony growth rate in this study, suggesting that CsHydr1 may affect surface tension to some extent, which is similar to other known hydrophobins.

However, not all hydrophobins are involved in spore superficial rodlet layers. Here, the subcellular localization of CsHydr1 was significantly different from some other fungal hydrophobins reported. In general, hydrophobins of filamentous fungi are secreted and cover the walls of spores and hyphae with a hydrophobic layer [25]. By their ability to aggregate to amphipathic membranes, they attach to the surface of the hydrophilic fungal cell wall, thereby exposing the hydrophobin layers to the outside, which can often be recognized by the formation of rodlets with scanning electron microscopy [26,45]. For example, six hydrophobins (Rod A, Dew A–E) in *Aspergillus nidulans* all localized on the conidiospore surface involved in hydrophobin rodlet formation and contributed to the hydrophobicity of the spore surface [54]. The absence of RodA caused the loss of the conidial rodlet structure, and other hydrophobins, such as Dew A–E, had minor effects on the Integrity of the rodlet layer [54]. Two hydrophobins, Hyd1 and Hyd2 in *Beauveria bassiana*, were also identified as localizing on the spore surface and were involved in spore coat rodlet layer formation [36]. However, we did not find a rodlet layer on the surface of wild-type spores and did not find a significant difference in the outside layer of the spore between the wild-type strain and Δ*CsHydr1*. Similar phenotypes were also found in *Botrytis cinerea*; neither the hydrophobin (Bhp1, Bhp2 and Bhp3) triple knockout mutants nor the wild-type conidia were covered with rodlet-shaped structures, and no differences were observed between them [55]. Furthermore, we found that CsHydr1 was significantly localized on the lipid droplet surface and interacted with the lipid-coated protein CsCap20 in *C. siamense*.

Some hydrophobins of plant/entomo-pathogenic fungi are involved in virulence. However, their roles in fungal virulence are different and remain to be understood. Knockout of *MPG1* in *M. grisea* results in impaired appressorium development and reduced infectivity [32,56]. The deletion of another hydrophobin gene, *MHP1*, in *M. grisea* also led to a loss of viability and a reduced capacity to infect and colonize a susceptible rice cultivar. In *B. bassiana*, the nonspecific hydrophobic interaction between the fungal spore coat hydrophobin and the insect epicuticle is involved in establishing the pathogenicity of the fungus [36]. *FgHyd2*, *FgHyd3* and *FgHyd4* are involved in the adhesion of conidia to wheat spikes during the early stages of the infection process, but *Fghyd1* and *Fghyd5* deletion mutants presented pathogenicity similar to WT [31].

This research confirmed the role of the pathogenicity function of CsHydr1 in *C. siamense*. However, it seems that the mechanism may be different from those of hydrophobins mentioned above. Our findings indicate that CsHydr1 interacts with the lipid droplet-coated protein CsCap20, localizes with intracellular lipid droplets in *C. siamense*, and influences lipid content. However, when CsCap20 was absent, some CsHydr1 was clearly observed in other parts (Figure 7c), we speculated that CsHydr1 is also localized on other organelles membrane lipid or dissociating lipid, and CsCap20 was helpful for the localization of CsHydr1 on lipid droplets, the interaction between CsHydr1 and CsCap20 is conducive to the formation and stability of lipid droplets in *C. siamense*. Previous studies have shown that the lipid droplet coating protein CsCap20 of *C. siamense* and its homologue Mpl1 in *M. anisopliae* affect the formation of lipid droplets in fungi and affect appressorium turgor and pathogenicity [22,41]. We speculate that CsHydr1 and CsCap20 jointly affect the formation of lipid droplets and the content of lipids, which in turn affect appressorium formation and turgor and further lead to reduced pathogenicity.

Thus, this study confirmed that the hydrophobin CsHydr1 interacts with the lipid droplet coating protein CsCap20 and localizes with lipid droplets and affects the intercellular lipid content of hyphae and spores.

## Data Availability

The data that support the findings of this study are available from the corresponding author upon reasonable request.

## Supplementary Materials

The following supporting information can be downloaded at: https://www.mdpi.com/article/10.3390/jof8090977/s1; Figure S1: Schematic representation of the targeted deletion of the *CsHydr1* gene by the homologous recombination method and molecular confirmation; Figure S2: Phylogenetic analysis of protein CsHydr1 and several known hydrophobin proteins in fungi; Table S1: All the primers used in this study.
