# Supplementary material for: The Colletotrichum siamense Hydrophobin CsHydr1 Interacts with the Lipid Droplet-Coating Protein CsCap20 and Regulates Lipid Metabolism and Virulence
Source: J Fungi (Basel). 2022 Sep 19;8(9):977. doi: 10.3390/jof8090977 (PMC9502314; doi:10.3390/jof8090977)
Supplement: Supplementary file 1 [file jof-08-00977-s001.zip › jof-1868853-supplementary.pdf]

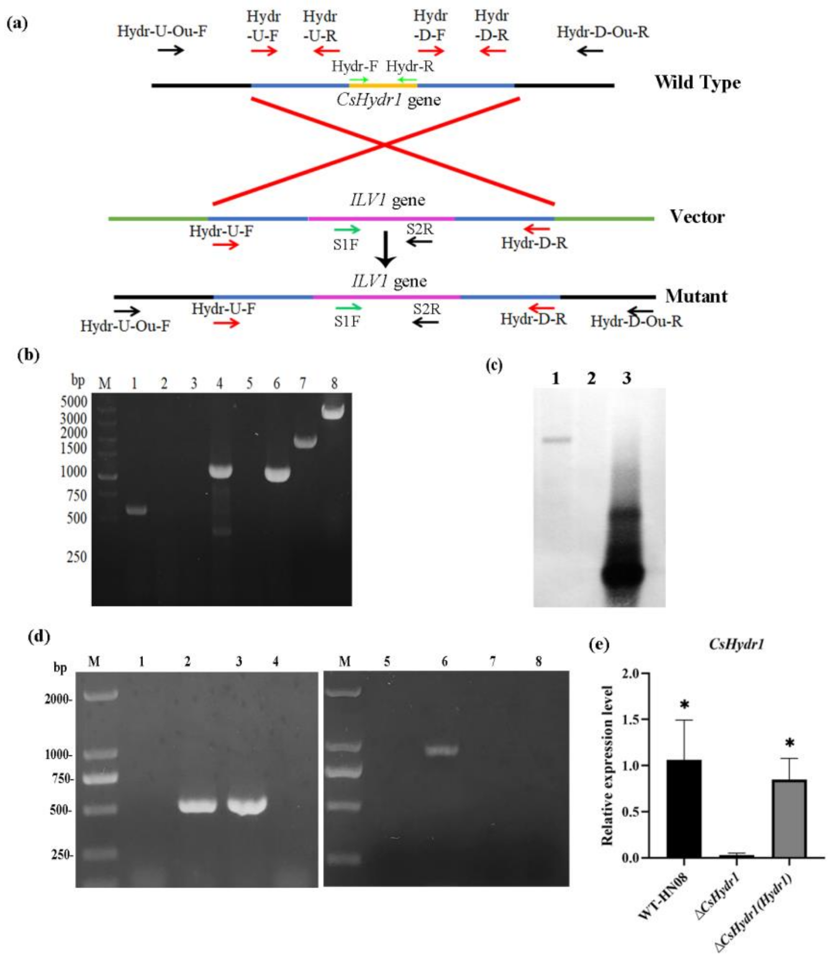

Figure S1. Schematic representation of the targeted deletion of the *CsHydr1* gene by the homologous recombination method and molecular confirmation. (**a**) Diagram describing the targeted gene deletion of *CsHydr1* and primers used for verification of the gene replacement event; (**b**) PCR verification of the *ΔCsHydr1* mutant. **(c)** Southern blot analysis confirming the *ILV1* gene number in mutant. Genomic DNA in *ΔCsHydr1* mutant (lane 1) and wild type (lane 2) was digested with *Eco*RI and probed with a *ILV1* coding sequence. Lane 3 was PCR fragment of *ILV1* showing as a positive control. **(d)** PCR verification of the strain *ΔCsHydr1(Hydr1)*. M: DNA DL2000 marker; Lanes 1-4 were product amplified by Hydr-F/Hydr-R from *ΔCsHydr1* mutant (lane 1), *ΔCsHydr1(Hydr1)* (lane 2), wild-type (lane 3) and ddH_2O (lane 4), respectively. Lanes 5-8 were product amplified by RP27-F/Hydr-R from *ΔCsHydr1* mutant (lane 5), *ΔCsHydr1(Hydr1)* (lane 6), wild-type (lane 7) and ddH_2O (lane 8), respectively. **(e)** Relate expression of *CsHydr1* determined by qRT-PCR in *ΔCsHydr1(Hydr1)* strains and wild type HN08. Expression levels were normalized using *ACT* expression levels as controls. Data were collected from three technical replicates. Error bars represent SD, * indicated significant differences within each measurement group(*p < 0.1, One-way Anova and Duncan's test) .

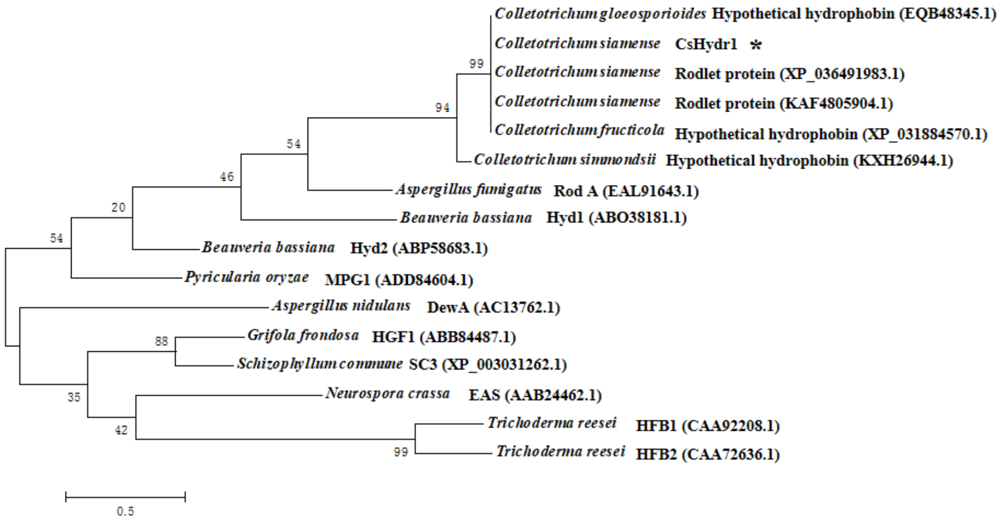

Figuer S2. Phylogenetic analysis of protein CsHydr1 and several known hydrophobin proteins in fungi. The phylogenetic tree was constructed with MEGA.6.0 using the maximum likelihood method. The CsHydr1 protein in this study was emphasized with a star.

Table S1 All the primers used in this study.

Primer	Sequence 5′-3′
hydr-F	CGCGGATCCGCGATGCGCTTCTCCGCTGCCACC
hydr-R	CGAGCTCGTTACAAAAGACCAGAGATGGGG
Mi-H1-F	CGGAATTCATGCGCTTCTCCGCTGCCAC
Mi-H1-R	CCCTCGAGCAAAAGACCAGAGATGGGGA
Mi-sp-H1-F	CGGAATTCATGGCTCCCGGCAACACTGCC
Mi-sp-H1-R	CCCTCGAGCAAAAGACCAGAGATGGGGA
pSUC2-F	TTCCTCGTCATTGTTCTCGTTC
pSUC2-R	GTCTATCGCTAGTTTCGTTTGTCTC
Hydr-U-F	CCCAAGCTTGGGGATAAATATCGATTGTAACTC
Hydr-U-R	CCGGAATTCCGGTTTCAAAGATAGAGTTGTTC
Hydr-D-F	GCTCTCACCGCGGATCCGATACCAACCGGATACATGCAG
Hydr-D-R	CTAGAACTAGTGGATCTTGCGACAATCGCAGGCGGTCG
Hydr-U-Ou-F	CGATGCGCTGTTCATGCGGCTAC
Hydr-D-Ou-R	GCACGATGAAGGTATCTATCC
S2F	GGCGGTGCTATCCTTCCCGTGTT
S1R	GTTCAACGCCGCCTTCCGACAAAAT
PXY203-Hydr1-F	TTTCGTAGGAACCCAATCTTCAAAATGCGCTTCTCCGCTGCCAC
PXY203-Hydr1-R	TTCGAATTTAGCAGCAGCGGTTTCTTTTTACAAAAGACCAGAGATGGGGA
RP27-F	TTTCGTAGGAACCCAATCTTCAAAATGGCCGACCCGTTTGCG
BK-Hydr1-F	CGGAATTCATGGCTCCCGGCAACACTGCC
BK-Hydr1-R	CGGGATCCCAAAAGACCAGAGATGGGGA
pGBKT7-F	GTGCGACATCATCATCGGAAG
pGBKT7-R	CCGGAATTAGCTTGGCTGC
AD-Cap20-F	CGGAATTCATGTCCAAAATGGCCCAAGTC
AD-Cap20-R	CGGGATCCGTTGTTGACCTTTTCGTTCACG
pGADT7-F	AATACCACTACAATGGATGATG
pGADT7-R	GAGATGGTGCACGATGCACAGT
pGEX-6p-Hydr1-F	CGGGATCCATGGCTCCCGGCAACACTGCC
pGEX-6p-Hydr1-R	CGGAATTCCAAAAGACCAGAGATGGGGA
pGEX-6p-1-F	GACCCAATGTGCCTGGATGC

pGEX-6p-1-R	CCGCTTACAGACAAGCTGTG
pET-Cap20-F	CGGGATCCATGTCCAAAATGGCCCAAGTC
pET-Cap20-R	CGGAATTCGTTGTTGACCTTTTCGTTCACG
pET32a-F	CTTCTGGTCTGGTGCCACGCGG
pET32a-R	GCTTCCTTTCGGGCTTTGTTAG
pFL21-Hydr1-F	CGACTCACTATAGGGCGAATTGGGTACTCAAATTGGGGTATTGAGC GATAATGCCACA
pFL21-Hydr1-R	CACCACCCCGGTGAACAGCTCCTCGCCCTTGCTCACCAGATCCTCTT CAGAGATGAGTTTCTGCTCCAAAAGACCAGAGATGGGGA
G418-F	CAAGATGGATTGCACGCAGG
G418-R	CGCTATGTCCTGATAGCGGT
Actin-F	TGGTATGGGCCAGAAGGA
Actin-R	GGACGGAAGGAGCGAACA
RT-Hydr1-F	GCTCCCGGCAACACTGCC
RT-Hydr1-R	CAGTGAGCTTCAGGCCAC
